# Cutting-Processed Single-Wall Carbon Nanotubes with Additional Edge Sites for Supercapacitor Electrodes

**DOI:** 10.3390/nano8070464

**Published:** 2018-06-26

**Authors:** Teayeop Kim, Mun Kyoung Kim, Yunjeong Park, Eunpa Kim, Jangho Kim, Wonhyoung Ryu, Hyung Mo Jeong, Kyunghoon Kim

**Affiliations:** 1 School of Mechanical Engineering, Sungkyunkwan University, Suwon 16419, Korea; skkty@skku.edu (T.K.); djy828@skku.edu (Y.P.); 2 Department of Materials Science & Engineering, Kangwon National University, Chuncheon 24341, Korea; munkyoungkim@kangwon.ac.kr; 3 Semiconductor R&D Center, Samsung Electronics, Hwaseong 18448, Korea; eunpa.kim@samsung.com; 4 Department of Rural and Biosystems Engineering, Chonnam National University, Gwangju 61186, Korea; rain2000@jnu.ac.kr; 5 Department of Mechanical Engineering, Yonsei University, Seoul 03722, Korea

**Keywords:** carbon nanotube, supercapacitor, functionalized CNT, energy storage

## Abstract

Carbon nanotubes are frequently selected for supercapacitors because of their major intrinsic properties of mechanical and chemical stability, in addition to their excellent electrical conductivity. However, electrodes using carbon nanotubes suffer from severe performance degradation by the phenomenon of re-stacking during fabrication, which hinders ion accessibility. In this study, short single-wall carbon nanotubes were further shortened by sonication-induced cutting to increase the proportion of edge sites. This longitudinally short structure preferentially exposes the active edge sites, leading to high capacitance during operation. Supercapacitors assembled using the shorter-cut nanotubes exhibit a 7-fold higher capacitance than those with pristine single-wall nanotubes while preserving other intrinsic properties of carbon nanotubes, including excellent cycle performance and rate capability. The unique structure suggests a design approach for achieving a high specific capacitance with those low-dimensional carbon materials that suffer from re-stacking during device fabrication.

## 1. Introduction

As the demands for renewable energy sources have grown in environmentally friendly industries, high-performance energy storage systems are required for applications ranging from mobile electronic instruments to electric vehicles and buildings. Various types of electrochemical energy storage devices have been developed to effectively match to the energy sources. As alternatives to traditional dielectric capacitors and batteries, supercapacitors (SCs) (also known as electrical double layer capacitors (EDLCs)) have gained great interest because of their high power density, long life, and high charge and discharge rates [1,2]. SCs store and release electrical energy based on the electrical double layers formed by electrostatic interactions between ions in the electrolyte and the electrodes [3]. This charge storage mechanism creates electrochemical energy without chemical/mechanical stress on the electrode materials, and introduces advantages such as high rate capabilities and long-term cycle stabilities [4]. The electrochemical performance of SCs is primarily affected by the active surface area of the electrode materials, since the specific capacitance is proportional to the number of ions interacting with the electrochemical surface area [5]. Thus, nanostructuring the electrode material to increase the number of active sites has been widely adopted to create high-performance electrodes for SCs. Recently, metal oxide- [6,7,8] and conductive polymer-based [9,10] materials for pseudocapacitors (which have a different mechanism from EDLCs, using a surface redox reaction) have been developed to resolve the insufficient capacitance issues [1] of carbon-based SCs. However, long-term stability issues are still a concern in pseudocapacitors since the redox reactions on the surface during ion storage induce a constraining stress in the electrodes by volume expansion [8,10]. To address these concerns and meet industrial requirements for complementary devices, carbon-based EDLC-type SCs have been a major target for energy storage systems. Low-dimensional (for example, 1- or 2-D) carbon nanomaterials such as carbon nanotubes [11,12,13], graphene [14,15,16], and porous activated carbon [17,18,19,20] have been studied for use in SC electrodes. The specific capacitance of a fully configured SC can be increased by large surface areas and high conductivities. Carbon nanotubes (CNTs) have attracted special interest as electrode materials for EDLCs because of their exceptional thermal [21], electrical [22], and mechanical [23] properties; moreover, the electrochemically active area is maximized by their unique 1-D structure [11,24,25]. Although CNTs theoretically increase the specific capacitance of EDLCs because of their unique structure, actual capacitances of CNT-based EDLCs only show moderately better or even poorer specific capacitances than SCs based on commercial activated carbon. Since re-stacking occurs during electrode fabrication from interaction between the CNTs, resulting in a reduction in exposed active surface, the performance of CNT electrodes in SCs can be severely degraded [26]. In this work, an ultrasonic-assisted cutting process (previously reported [27]) is used to prepare cut single-wall CNTs (c-SWNTs) for SC electrodes. The cross-section of the cut edge in c-SWNTs can provide additional active sites for attaching electrolyte ions during SC operation. Moreover, the active sites of c-SWNTs are easily exposed even after re-stacking because of the short length of c-SWNTs. By this simple structural modification, the electrochemical performance of c-SWNT electrodes shows a remarkable improvement in comparison to conventional SWNT electrodes. The as-prepared c-SWNT electrode exhibits a 7-fold higher specific capacitance than SWNT electrodes and excellent rate capabilities with 100% capacity retention after 2500 cycles at 5 A g^−1^.

## 2. Materials and Methods

### 2.1. Chemicals and Materials

Chemicals: All reagents, unless otherwise stated, were obtained from commercial sources (NanoLab Inc, Waltham, MA, USA; Thermo Fisher Scientific Chemicals, MA, USA; Sigma-Aldrich, Darmstadt, Germany; Duksan, Ansan, Korea) and were used without further purification. Specifically, SWNTs (purity > 95%) with an average diameter of 1.5 nm were purchased from NanoLab Inc. (Waltham, MA, USA). Carbon Black (Super P, Conductive 99+% metal basis) was purchased from MTI Corp, CA, USA. Poly(vinylidene fluoride) (PVDF, average *M_w_*~534,000 by GPC, powder), 1-methyl-2-pyrrolidinone (NMP, ReagentPlus^®^, 99%), sodium sulfate (Na_2_SO_4_, ReagentPlus^®^, ≥99.0%), and nickel foil (thickness 0.1 mm, 99.98% trace metals basis) were purchased from Sigma-Aldrich. Distilled water was purchased from Duksan. All chemicals were used as received in air.

### 2.2. Fabrication of Short-Cut Single-Wall Carbon Nanotubes

**Figure 1:**
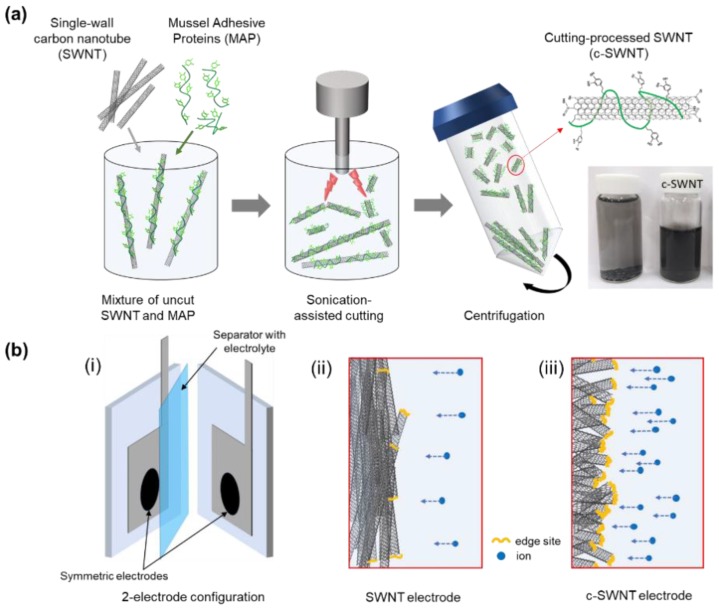
(**a**) The schematic of the synthesis process of short-cut SWNT (c-SWNT) and photographs of carbon nanotube suspension before and after cutting process (**b**) Schematic illustration of the supercapacitor structure (i) and electrodes using (ii) SWNTs and (iii) c-SWNTs. The short structure of c-SWNT preferentially exposes the active edge sites during operation.

As-purchased SWNTs (5 mg) and lyophilized mussel adhesive protein (MAP, *M_w_*~25,000) powder (15 mg) were added to a glass vial and mixed with 20 mL of DI water. MAP was added to enhance dispersion of CNTs in aqueous solution. Prior to cutting process, the aqueous mixture of SWNTs and MAP was bath-sonicated for 1 h to obtain a homogeneous dispersion. For the sonication-assisted cutting process, 110 W pulses were applied (3 s on; 1 s off) using a VC505 horn-type sonicator (Sonics, CT, USA) for 10 h (Figure 1a). During sonication, the vial was held on a metal rack and immersed in an ice bath to remove the ultrasonic energy absorbed. To separate the cut carbon nanotubes from the uncut nanotubes, cutting-processed mixture solution was centrifuged at 4000 rpm for 1 h (1580R, Labogene, Seoul, Korea) and then c-SWNT suspension stabilized by MAP were obtained. Finally, the c-SWNT solution produced was freeze-dried for Fourier Transform Infrared (FT-IR) spectroscopy and Raman analyses.

### 2.3. Material Characterization

The Raman spectra were scanned by an XperRam Compact confocal Raman spectrometer (Nanobase, Seoul, Korea) with a laser wavelength of 405 nm. For the preparation of the samples for Raman spectra, SWNTs and c-SWNTs were dispersed in ethanol by bath sonication, dropped onto glass slides, and dried. For FT-IR spectroscopy analysis, the SWNT and freeze-dried c-SWNT powders were deposited in a KBr pellet. The FT-IR spectra in the 400–4000 cm^−1^ range were measured using an IFS-66/S FT-IR spectrometer (Bruker, Ettlingen, Germany) with 1.9 cm^−1^ resolution. High-resolution transmission electron microscopy (JEM-3010, JEOL, Tokyo, Japan) was used to investigate nanostructures of SWNT and c-SWNT.

### 2.4. Electrochemical Measurements

The electrochemical measurements of SWNT and c-SWNT electrodes were conducted in a two-electrode system (Figure 1b). A slurry was first prepared by dissolving samples and polyvinylidene fluoride (PVDF) (w:w = 9:1) in N-methylpyrrolidone (NMP). The slurry was cast onto nickel foil and dried in a vacuum oven at 60 °C for 12 h. For aqueous electrolyte devices, two pieces of the c-SWNTs cast onto substrates were placed on Teflon plates; platinum leads contacted the back sides of the substrates for potentiostat (Biologic VMP3) connections; the two Teflon plates were assembled into a sandwich with a separator (Whatman 8 µm filter paper) between them. The cell assemblies were wrapped with parafilm, then dipped in the electrolyte solutions (1 M Na_2_SO_4_). For all the cell types, the active area overlapped by both sample films was 1 cm^2^. Typical mass loadings for measurements were ~0.3 mg cm^−2^.

In the galvanostatic data, the IR drop at the upper cut-off potential and the slope in the discharge curve are used to obtain the average power and energy densities. The capacitance (*C_t_*) was calculated using the following relationship

*C_t_* = *it*/∆*V* (1)

The specific capacitance is defined as the capacitance of a single electrode per unit weight. In this work, an symmetric capacitor consists of a series of electrodes with half the total electrode weight. Therefore, the specific capacitance (*C_s_*) is calculated by using the following equation.

*C_s_* = 4 *C_t_*/*m* (2)

where *i* is the applied current (A), *t* is the discharge time (s), *m* is the total mass (g) of active materials in both electrodes, and Δ*V* is the potential difference (V).

## 3. Results and Discussion

Short and edge-site activated SWNTs were prepared by the protein-assisted sonication cutting process and fabricated into the electrodes to obtain an EDLC-type SC configuration (Figure 1). As described in Figure 1, the active edge-sites of c-SWNTs become part of the effective surface area for storing ions from the electrolyte, leading to the high specific capacitances of the SCs. To compare the microstructure and chemical composition of c-SWNT and SWNT, various analysis techniques were used, including High-resolution transmission electron microscopy (HR-TEM), Raman spectroscopy, and FT-IR.

**Figure 2:**
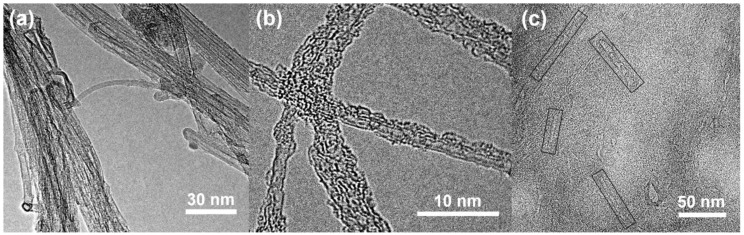
The HR-TEM images of (**a**) as-purchased SWNTs; and (**b**,**c**) functionalized c-SWNTs after sonication cutting.

The HR-TEM images show the morphologies of c-SWNTs and SWNTs (Figure 2). The as-purchased SWNTs (Figure 2a) show bundles of SWNTs, mainly composed of SWNTs. However, after the sonication-assisted cutting process, the c-SWNTs (Figure 2b) show unbundled individual short CNTs with a MAP-coated sidewall. Isolated c-SWNTs of short length (Figure 2c) are seen with additional edge sites induced by the sonication process.

**Figure 3:**
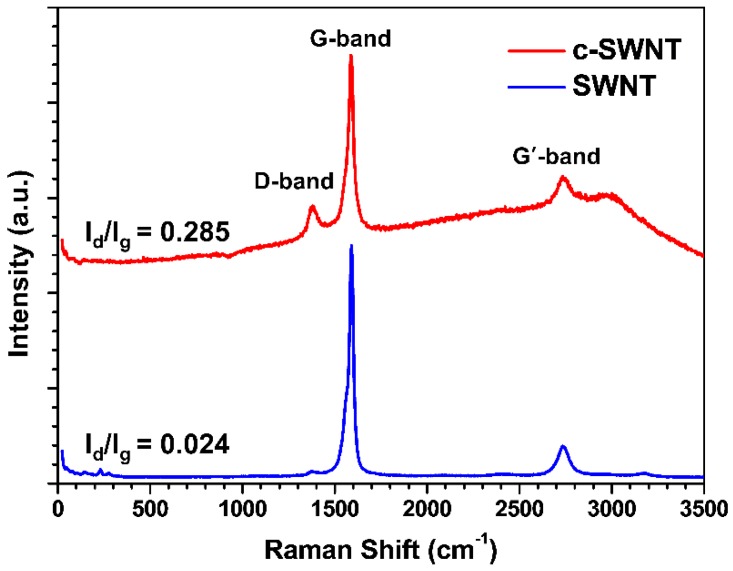
The Raman spectra of SWNT and c-SWNT.

In carbon-based materials such as SWCNTs, Raman spectroscopy is a powerful tool to recognize ordered and disordered (damaged) structures [28]. As shown in Figure 3, the peak corresponding to the D band around 1353 cm^−1^ is increased in the c-SWNT samples, demonstrating that tailoring the SWNTs to shorter lengths generates the defects seen by the D band. The ratio of the D/G bands (*I_D_/I_G_*) indicates the degree of disorder, related to the shortened SWNTs with edge-site defects. The *I_D_/I_G_* intensity ratio in c-SWNT (0.285) is higher than in the SWNTs (0.024). While both SWNT and c-SWNT show a G’ band presenting defect free sp^2^ carbon [29], this result indicates that exposing the functionalized edge-site by a cutting process introduces a relatively higher level of disordering compared to pristine SWNTs.

**Figure 4:**
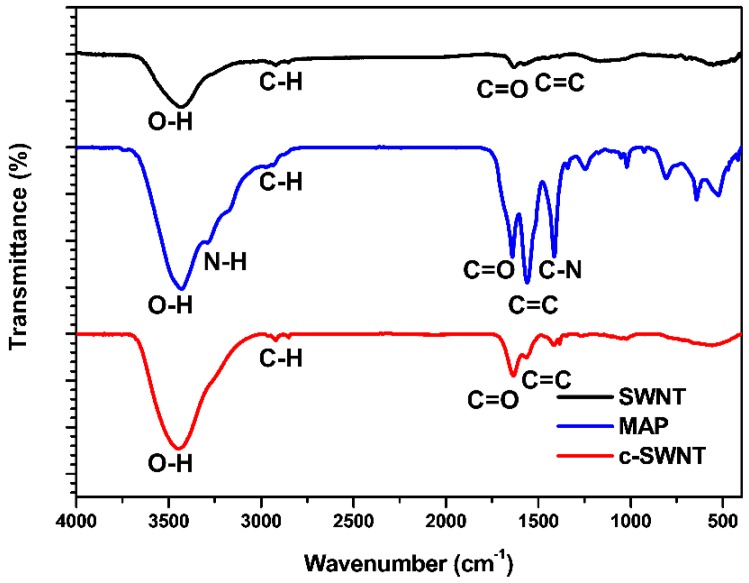
The FT-IR spectra of SWNT, MAP, and c-SWNT.

During the cutting process, it is expected that the surface functionalities of SWNTs will be changed. The FT-IR spectra of SWNT, MAP, and c-SWNTs exhibited in Figure 4 demonstrate the functionalization of edge sites and sidewalls that occurs in the sonication-assisted cutting process. The FT-IR spectra of pristine SWNT show a wide absorption band around ~3500 cm^−1^ (O–H) and weaker absorption peaks at ~2900 cm^−1^ (C–H), ~1740 cm^−1^ (C=O), and ~1600 cm^−1^ (C=C). The weak absorption peaks around ~3500 and ~1740 cm^−1^, corresponding to hydroxyl and carboxyl groups, respectively, indicate low functionality of pristine SWNT [30,31]. In comparison, the c-SWNT spectrum shows larger absorption peaks than SWNT at ~3500 and ~1740 cm^−1^, and the peaks at 2900 and 1660 cm^−1^ (corresponding to graphitic structure) show similar intensity. This is attributed to both presence of MAP and the formation of additional functional groups on c-SWNTs. The absorption peak of C–N (~1385 cm^−1^) in the c-SWNTs sample is much lower than in the sample with MAP alone. It indicates that additional hydroxyl and carboxylate groups at edge sites in the c-SWNTs were produced by the sonication-assisted cutting process [30,31]. It makes the absorption peak intensity of increased functional group relatively stronger than that of the C–N bond as shown in the FT-IR spectrum of c-SWNT.

**Figure 5:**
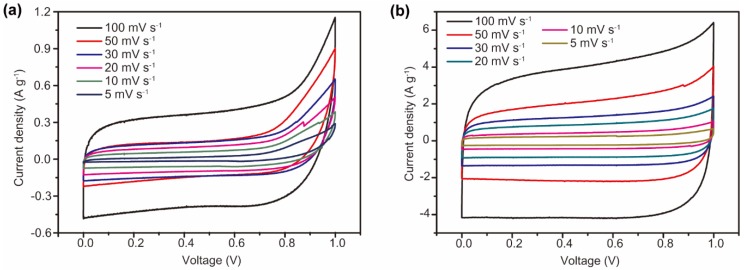
The CV curves of (**a**) SWNT electrodes and (**b**) c-SWNT electrodes at various scan rates from 1 to 100 mV s^−1^. Note the different current scales in (**a**,**b**).

To confirm the morphological advantages in SC performance arising from c-SWNT electrodes, the electrochemical properties of c-SWNT and SWNT electrodes were evaluated using a two-electrode configuration with an aqueous electrolyte. Electrodes with the same mass loading were assembled in sandwich type SC devices with a 1 M Na_2_SO_4_ aqueous solution (Figure 1b). Figure 5 shows the cyclic voltammetry curves of the SWNT and c-SWNT electrodes, taken in the range 0 to 1 V at scan rates of 5 to 100 mV s^−1^. Generally, the SC devices using SWNTs and c-SWNTs show almost rectangular cylclic voltammetry (CV) curves, corresponding to EDLC-type SC devices operating by electrostatic adsorption of ions. With the SWNTs, the shape of the CV curves is distorted by the overpotential near 1 V when charging at all the scan rates used. In contrast, the c-SWNT SCs present rectangular CV curves through the range of measurement conditions, without overpotentials even in the high potential region, indicating that electrostatic absorption of ions is increased by the additional exposed active sites. It is also significant that the area of the CV curves for the c-SWNT SCs is remarkably enlarged (note the different current scales in Figure 5) compared to the SWNT SCs; in general, the areas of CV curves indicate the specific capacitance of the two-electrode SC devices.

**Figure 6:**
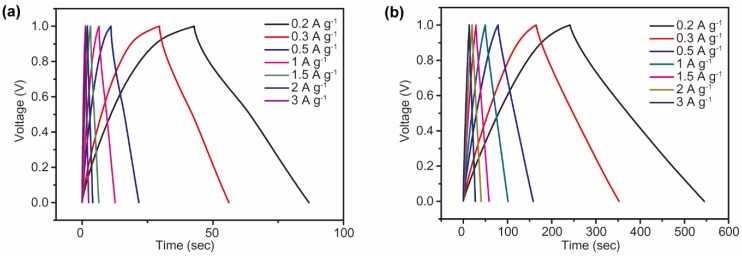
The charge/discharge profiles of (**a**) SWNT electrodes and (**b**) c-SWNT electrodes at current densities from 0.5 to 3 A g^−1^.

Galvanostatic charge and discharge tests of SWNT and c-SWNT electrodes were performed at current densities from 0.2 to 30 A g^−1^. For a constant current density between 0.2 and 3 A g^−1^, the SWNT and c-SWNT electrodes exhibit a linear charge/discharge profile, without a plateau signifying the redox reactions between materials and ions (Figure 6). As expected from the charge/discharge behavior displayed in the CV curves (Figure 5), an overvoltage is seen in the discharge profiles of the SWNT electrodes, indicating the capacity degradation from the re-stacking of electrode materials (Figure 6a). On the other hand, the c-SWNT electrodes show stable charging behavior and a significantly increased discharge time, indicating the enhanced specific capacity of c-SWNT electrodes resulting from the increased number of active edge sites (Figure 6b). The charge/discharge profiles of the c-SWNT electrodes again correspond to the behavior seen in the CV curves of Figure 5.

**Figure 7:**
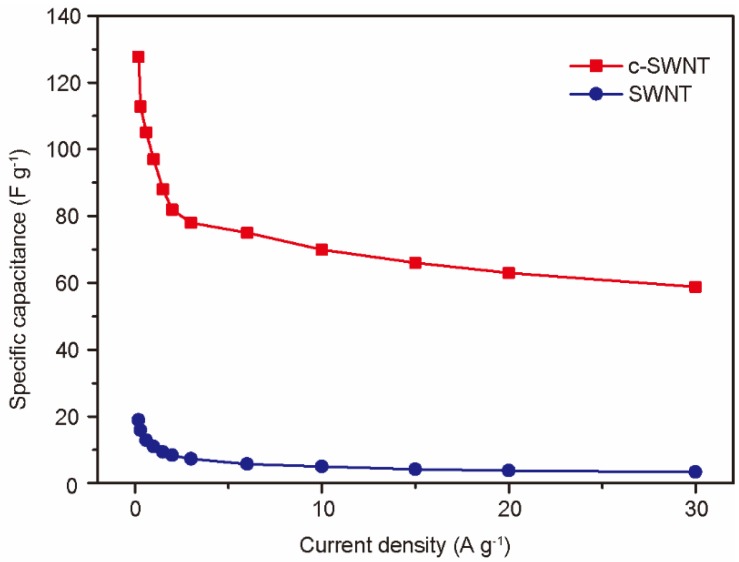
The rate capabilities of SWNT and c-SWNT electrodes at current densities from 0.2 to 30 A g^−1^.

To further evaluate the performance of devices at realistic scan rates, the rate capabilities of SWNT and c-SWNT were investigated by gravimetric measurements at current densities from 0.2 to 30 A g^−1^ (Figure 7). The highest specific capacitances of SWNT and c-SWNT electrodes were observed to be 18.94 and 127.68 F g^−1^, respectively, at 0.2 A g^−1^. Even at the highest current density used (30 A g^−1^), the specific capacitance of the c-SWNT electrodes is 58.8 F g^−1^, while the SWNT electrodes show a severely degraded capacitance of 3.3 F g^−1^. The defects or edge sites of the nanocarbon materials stimulate ion adsorption by the relatively active surface energy. It is notable that the number of defects and edge sites increased during the c-SWNT cutting process, leading to the dramatically enhanced specific capacitance of SCs despite the re-stacking problems of SWNTs, as presented in Figure 1b.

**Figure 8:**
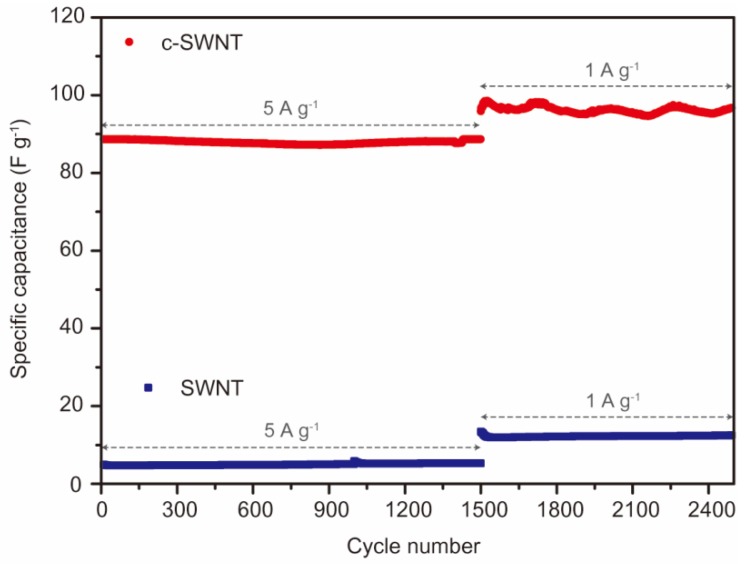
The cycle retention of SWNT (blue) and c-SWNT (red) electrodes at a current density. The first 1500 cycles were performed at 5 A g^−1^, followed by an additional 1000 cycles at 1 A g^−1^.

Typically, with carbon-based electrodes, the capacity retention of SCs is exceptionally stable over thousands of charge/discharge cycles. SCs specifically using CNTs also generally show stable cycling performance. Figure 8 shows the cycle stabilities of SWNT and c-SWNT SCs, tested at a current density of 5 A g^−1^ for 1500 cycles and then at 1 A g^−1^ over a further 1000 cycles. It is not surprising that SWNT SCs exhibit capacitance retention over 2500 cycles without degradation. With the c-SWNTs, the SCs show an excellent long-term stability that is comparable to the SWNT SCs. It should be noted that the increased specific capacitance of c-SWNT SCs at these current densities is another robust property of carbon materials, and survives the additional fabrication step for c-SWNTs. The SWNT cutting process exposes additional active sites and enhances the electrochemical performance, overcoming the capacitance loss resulting from the re-stacking phenomenon during electrode fabrication. Moreover, the superior intrinsic properties of CNTs are maintained through the cutting process.

## 4. Conclusions

In conclusion, shortened c-SWNTs with additional active sites from the exposed tube edges have been fabricated from SWNTs using a sonication-assisted cutting process for high performance SC electrodes. Normally, the CNT-based SCs show a degraded specific capacitance, since the ion adsorption is interrupted by the re-stacking of nanotubes, which induces blocking of the active surfaces. Meanwhile, the cutting process of SWNTs, resulting in c-SWNTs, introduces additional active edge sites for ion adsorption; these lead to improved electrochemical properties by counteracting the re-stacking effects. Moreover, the original properties of carbon materials are maintained in c-SWNT, introducing long-term stability. The SCs using c-SWNTs exhibit a 7-fold higher specific capacitance (127 F g^−1^) compared to SCs using SWNTs, a high rate capability for current densities from 0.2 to 30 A g^−1^, and an excellent cycle stability (100% capacitance retention after 2500 cycles). This work suggests that sonication-assisted cutting is a simple strategy to enhance the electrochemical properties of CNT-based electrodes for SCs.
